# Hernia with spermatocele, cord and testis inside sac: Case report

**DOI:** 10.1016/j.ijscr.2018.11.031

**Published:** 2018-11-22

**Authors:** Imtiaz Wani

**Affiliations:** Department of Surgery, DHS Hospitals, Srinagar, 190009, Kashmir, India

**Keywords:** IEA, Inferior epigastric artery, USG, Ultrasonography, Hernia, Spermatocele, Testis, Cord

## Abstract

Introduction: Giant spermatocele,testis and spermatic cord as content of sac in an indirect hernia is extremely rare. These are congenital in.nature.

Case Report: A 61 year old male was diagnosed as a case of giant spermatocele.Exploration documented giant spermatocele and an indirect inguinal hernia of complete type with the testicle, cord and spermatocele as a content of herrnia sac Excision of spermatocele with placement of cord outside sac was done.This is a first kind of case report with new type of hernia reported with cord as content.

Conclusion: Giant spermatocele is to be excised.Testis and spermatic cord inside hernia sac have to be made extrasacal to prevent recurrence.

## Highlights

• Extremely rare.
• First Kind of case series in Literature.
• Giant Spermatocele extremely Rare.
• Cord and testis inside hernia sac never reported.

## 1 Introduction

Giant spermatocele is a rare to see and can be unilateral or bilateral [1]. Spermatocele most frequently occur in the fourth and fifth decades of life in males [2]. Both indirect inguinal hernia and spermatocele are considered to be of congenital origin.Occurrence of testis,spermatic cord and spermatocele inside hernia sac is extremely rare. The work has been reported in line with the SCARE criteria

## 2 Case presentation

**Fig. 1:**
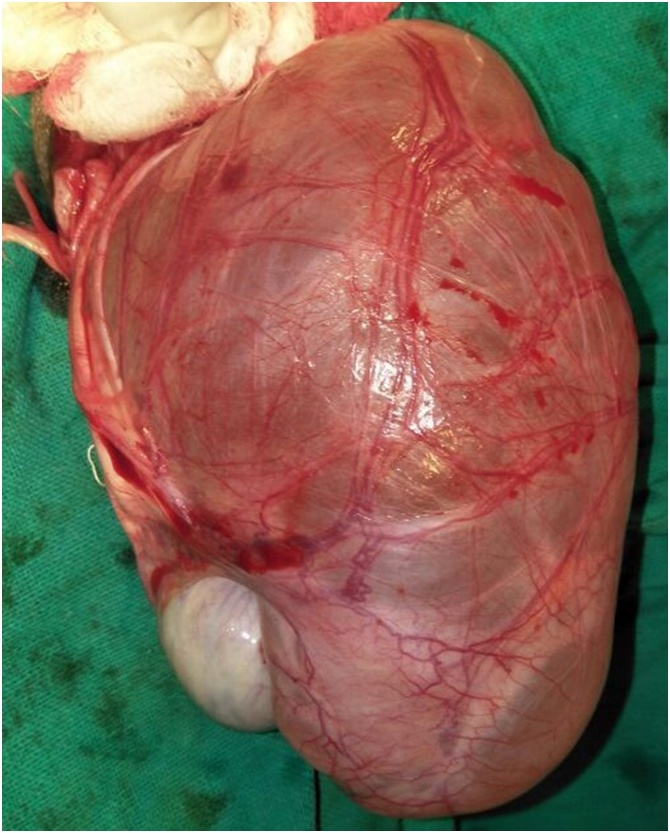
Showing giant hydrocele.

**Fig. 2:**
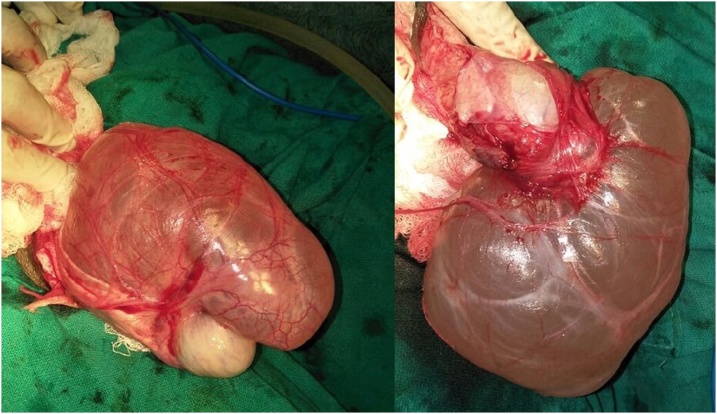
Showing giant spermatcele inside sac.

**Fig. 3:**
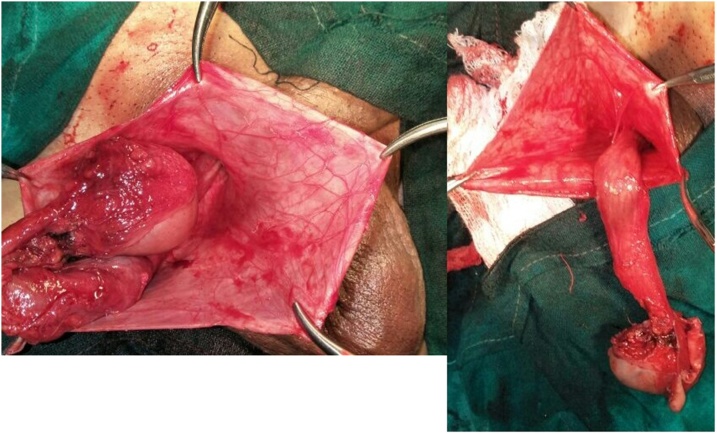
Showing cord and testis inside sac.

**Fig. 4:**
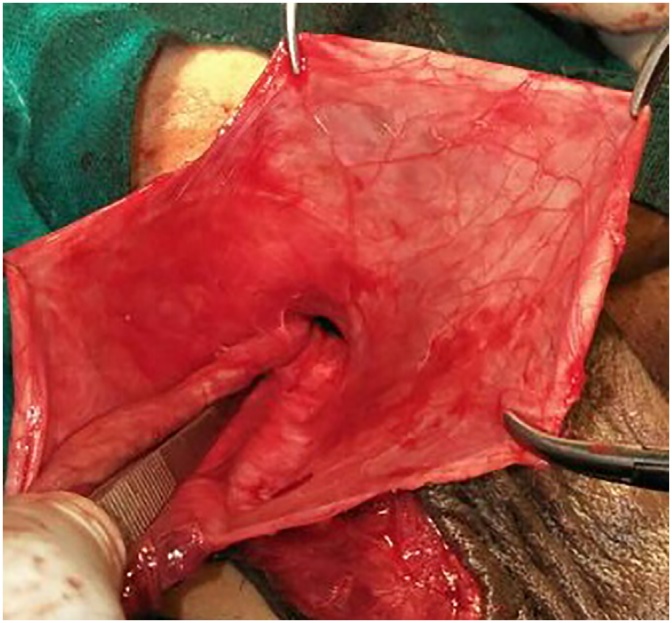
Showing testis,cord inside sac traversing inguinal canal.

A 61 year old male presented with painless swelling of right scrotum of 10 years duration. There was no history of any trauma,orchitis or any inguino-scrotal surgery. Systemic examination was normal. Local examination revealed a large swelling soft, non tender, non reducible with no expansile cough impulse having free overlying skin reaching base of scrotum. Transillumination test was positive. Scrotal USG showed 371 ml fluid filled unilocular cyst suggestive of the spermatocele. Exploration of scrotum revealed a large unilocular cyst arising from rete testis and partially covered by a thin membranous sheath (Fig. 1, Fig. 2) This giant spermatocele was dissected from testicle and released of sac. Testicle and spermatic cord were enclosed in this thin sac like structure and traced to deep ring going into peritoneal cavity (Fig. 3). Palpation of deep ring admitted index finger with omentum at deep ring Diagnosis of an indirect hernia of a complete type with spermatocele, cord, testicle and omentum as a contents was made. Sac was dissected upto deep ring, incised laterally and cord with testicle kept out of sac (Extrasacal) (Fig. 4). Twisting of incised sac with high ligation of sac done, released into peritoneal cavity. Histopathology confirmed diagnosis of spermatocele and that of sac of hernia showed fibrous type. Follow up period was uneventful.

## 3 Discussion

Giant spermatocele is rare to see. This usually presents as a slowly growing painlessunilocular swellingi [2]. Development is of idiopathic nature but may be of congenital origin or results from epididymitis, trauma, inguino-scrotal surgery or vasectomy, these leading to scarring, obstruction of proximal efferent ducts and may form a spermatocele [3]. Males whose mothers given the drug diethylstilbestrol (DES) during pregnancy to prevent miscarriage and other pregnancy complications have a increased risk of spermatocele occurrence. Spermatocele is usually located on the superior aspect of the testicle. This is distinct from hydrocele, which envelops the testis on the anterior and lateral surfaces the testicle but does not displace it. Histopathology shows a lining of a single layer of cuboid, often ciliated epithelium and has thin wall of fibromuscular soft tissue. The cyst contains barley -colored fluid being proteinaceous fluid with spermatozoa, cholesterol clefts and exhibiting foreign body giant cell reaction.

An Indirect inguinal hernia is usually a congenital. A patent processus vaginalis and increased cumulative mechanical exposure are risk factors for indirect inguinal hernia occurrence [4]. Aberrant hernia has been suggested to occur due defective regulatory mechanism of hormones, peptides from the genitofemoral nerve and insufficient release of calcitonin gene-related peptide that have an effect on testicular descent [5]. In this case, spermatic cord and testis invested by processus vaginalis in embryogenesis failed to disappear altogether after descent. The hernia was itself being complete with internal ring admitting index finger and sac spreading like cone. Arsalan et al. [5] reported an adult case with cryptorchidism in which testis and spermatic cord constitute a component of the indirect inguinal hernia sac but in the present case testis and spermatic cord were content without any cryptochidism. Spermatocele with testis and cord is to be made extrasacal after incising lateral wall of sac and transfixed at deep ring to prevent recurrence, omentocele or enterocele

## 4 Conclusion

Giant spermatocele, coexistence with indirect hernia with cord with testis inside hernia sac is never reported. Giant spermatocele is to be excised. Testis and spermatic cord inside hernia sac have to be made extrasacal to prevent recurrence.

## Conflictsofinterest

None.

## Sources of funding

None.

## Ethical approval

The publication of my article, if the study is exempt from ethnical approval in my institution.

## Consent

Written and signed consent to publish a case report obtained from patient.

## Author contribution

IW made study concept or design, data collection, data analysis or interpretation, writing the paper.

## Registration of research studies

NA.

## Guarantor

Imtiaz Wani is guarantor and accept full responsibility for the work and the conduct of the study, had access to the data, and controlled the decision to publish.

## References

[1] Basar H., Baydar S., Boyunaga H., Yilmaz E. (2003). Primary bilateral spermatocele. Int. J. Urol..

[2] Yagi H., Igawa M., Shiina H., Shigeno K., Yoneda T., Wada Y. (2001). Multilocular spermatocele: a case report. Int. Urol. Nephrol..

[3] Yeh H.C., Wang C.J., Liu C.C., Wu W.J., Chou Y.H., Huang C.H. (2007). Giant spermatocele mimicking hydrocele: a case report. Kaohsiung J. Med. Sci..

[4] Öberg S., Andresen K., Rosenberg J. (2017). Etiology of inguinal hernias: a comprehensive review. Front. Surg..

[5] Arslan Y., Karaman K., Altintoprak F., Kahyaoglu Z., Zengin I., Uzunoglu M.Y., DemirH (2014). Indirect inguinal hernia sac containing testis and spermatic cord in an adultpatient with cryptorchidism. J. Surg. Case Rep..

